# Effect of fluoride toothpastes on enamel demineralization

**DOI:** 10.1186/1472-6831-6-8

**Published:** 2006-06-15

**Authors:** Wolfgang H Arnold, Andreas Dorow, Stephanie Langenhorst, Zeno Gintner, Jolan Bánóczy, Peter Gaengler

**Affiliations:** 1 Faculty of Dental Medicine, University of Witten/Herdecke, Witten, Germany; 2 Faculty of Dentistry, Semmelweis University, Budapest, Hungary

## Abstract

**Background:**

It was the aim of this study to investigate the effect of four different toothpastes with differing fluoride compounds on enamel remineralization.

**Methods:**

A 3 × 3 mm window on the enamel surface of 90 human premolars was demineralized in a hydroxyethylcellulose solution at pH 4.8. The teeth were divided into 6 groups and the lower half of the window was covered with varnish serving as control. The teeth were immersed in a toothpaste slurry containing: placebo tooth paste (group 1); remineralization solution (group 2); Elmex Anticaries (group 3); Elmex Sensitive (group 4); Blend-a-med Complete (group 5) and Colgate GRF (group 6). Ten teeth of each group were used for the determination of the F^- ^content in the superficial enamel layer and acid solubility of enamel expressed in soluble phosphorus. Of 6 teeth of each group serial sections were cut and investigated with polarization light microscopy (PLM) and quantitative energy dispersive X-ray analysis (EDX).

**Results:**

The PLM results showed an increased remineralization of the lesion body in the Elmex Anticaries, Elmex Sensitive and Colgate GRF group but not in the Blend-a-med group. A statistically significant higher Ca content was found in the Elmex Anticaries group. The fluoride content in the superficial enamel layer was significantly increased in both Elmex groups and the Blend-a-med group. Phosphorus solubility was significantly decreased in both Elmex groups and the Blend-a-med group.

**Conclusion:**

It can be concluded that amine fluoride compounds in toothpastes result in a clearly marked remineralization of caries like enamel lesions followed by sodium fluoride and sodium monofluorophosphate formulations.

## Background

Today, the caries preventive effect of fluoride is without any doubt. Among caries preventive protocols fluoride containing dentifrices are well accepted. For the caries preventive effect the bioavailability of fluoride is of importance [1]. Bioavailability of fluoride is dependent from the solubility of the fluoride containing compound and from the adhesion of the fluoride compound to the surface [2]. In dentifrices different fluoride formulations are used as carrier for fluoride ions of which the most frequent are sodium fluoride, sodium monofluorophosphate and amine fluoride. Contradictory results have been published about the effectiveness of the different fluoride formulations in toothpastes [3-5]. In a recent study it has been shown that sodium fluoride (NaF) is more effective in caries prevention than sodium monofluorophosphate but this study did not include amine fluoride [5]. The effect of amine fluoride on enamel remineralization has been studied in experimental [6,7] and clinical [8,9] investigations and showed similar effects of amine fluoride and NaF.

A lot of attention has been paid to the amount of fluoride in dentifrices [10-13]. Most studies showed that already low concentrations of salivary fluoride effect enamel demineralization and remineralization. Salivary fluoride levels decrease with the time after topical application with a fluoride dentifrice [14-16]. For the effectiveness of fluoride over periods longer than the brushing and the following salivary clearance, fluoride needs to be deposited and slowly released [17]. Together with Ca ions, fluoride forms the calcium fluoride (CaF_2_) compound which slowly releases fluoride and maintains the salivary fluoride level. The different solubility of NaF, sodium monofluorophosphate and amine fluoride may result in different amounts of CaF_2 _formation and hence influence the bioavailability of fluoride in saliva and the demineralization and remineralization potential of enamel. For amine fluoride, being a kationic tenside, the surface tension has also been discussed being responsible for the caries protective effect [2,18-20].

It is the advantage of morphological methods to directly demonstrate the effects of fluoride on demineralization and remineralization [7,21,22]. Furthermore with quantitative EDX element analysis it is possible to quantify these processes. It was therefore the aim of this study to investigate possible differences in enamel remineralization due to different fluoride compounds in dentifrices with polarization light microscopy and with quantitative EDX element analysis.

## Methods

Ninety for orthodontical reasons extracted caries free premolars were covered with varnish leaving a 3 × 3 mm window and divided into 6 groups of 15 teeth in each group. They were kept in a demineralizing gel (hydroxyethylcellulose) at pH 4.8 for 50 days. After demineralization the lower half of the window was also covered with varnish serving as positive control. The teeth of each group were then incubated in different toothpaste slurries, control medium or remineralization solution for 48 hours which simulates 2 years of tooth brushing 2 times for 2 minutes per day [23,24]. The remineralization solution contained potassium chloride (KCl 1 mmol/l), sodium acetat (0,2 mol/l), calcium chloride (CaCl_2 _150 mmol/l) and potassium hydrogen phosphate (KH_2_PO_4 _90 mmol/l). Incubation media are summarized in Table 1.

**Table 1:** List of incubation media.

Incubation medium	Manufacturer	Fluoride	Fluoride content (mg/l)	pH
Group 1 Placebo tooth paste	Ø	Ø	Ø	6.3
Group 2 Remineralization solution	Ø	Ø	Ø	7.4
Group 3 Elmex Anticaries	GABA AG	Amine fluoride	1250	4.7
Group 4 Elmex Sensitive	GABA AG	Amine fluoride	1400	5.1
Group 5 Blend-a-med Complete	Procter and Gamble	Sodium fluoride	1450	7.6
Group 6 Colgate GRF	Colgate Palmolive	Sodium monofluorophosphate	1450	7.0

After treatment with slurries 30 teeth were embedded in Technovit 9100 (Kulzer, Weinheim, Germany) and serial sections through the lesions with a thickness of 80 μm were cut using a saw microtome (Leica 1600, Bensheim, Germany). All sections were investigated with polarization light microscopy (PLM) and the birefringence of the lesions was categorized according to their morphological appearance, and to each category a numerical index number was assigned as follows: no lesion (1), single porosities (2), interrupted lesion band (3), inhomogeneous lesion (4) and completely homogeneous lesion (5). The numerical values were statistically compared using the nonparametric Mann-Whitney test.

Three sections of each lesion were then coated with carbon and examined with a scanning electron microscope (Philips XL 30 FEG, Eindhooven, The Netherlands) at 20 kV using the backscattered electron detector. In each experimental and control window of the different teeth 3 spot measurements (spot size 2 nm) were carried out on the enamel surface, within the body of the lesion and on surrounding sound enamel, resulting in a total number of 9 measuring points per window. Element content in weight % of Ca, P, C, and F was measured with energy dispersive X-ray analysis (EDX) with a S-UTW detector (EDAX INC, Mahwah, NJ, USA). The count rate of the EDX detector was between 1800 and 2000 counts per second with a dead time of 30 %. Measuring time was 30 s (live seconds) with a resolution of 135.8 eV and an amplification time of 100 μs. Line scans through the lesions were made at 256 points with a dwell time of 1000 ms and amplification time of 100 ms. The values of the spot measurements were statistically evaluated using the nonparametric ANOVA test for repeated measurements. As 3 calculations were made on the same set of data, the Bonferroni correction for p = 0.05 was p = 0.016.

On ten teeth of each group incubated with slurries (n = 60), enamel etch samples were conducted on control windows and on experimental windows by etching an isolated round surface of the teeth for 1 min with 4 μl of 0.5 % perchloric acid [25,26]. The enamel samples were analyzed for fluoride using the ion-specific Orion-9609 electrode. The resistance to acids of dental enamel was measured in terms of the amount of dissolved phosphorus resulting from the etching process. Phosphorus was determined photometrical by using flow injection analysis. The results were evaluated with Student's paired t-test.

This study was conducted with the approval of the Ethical Committee of the University of Witten/Herdecke.

## Results

**Figure 1:**
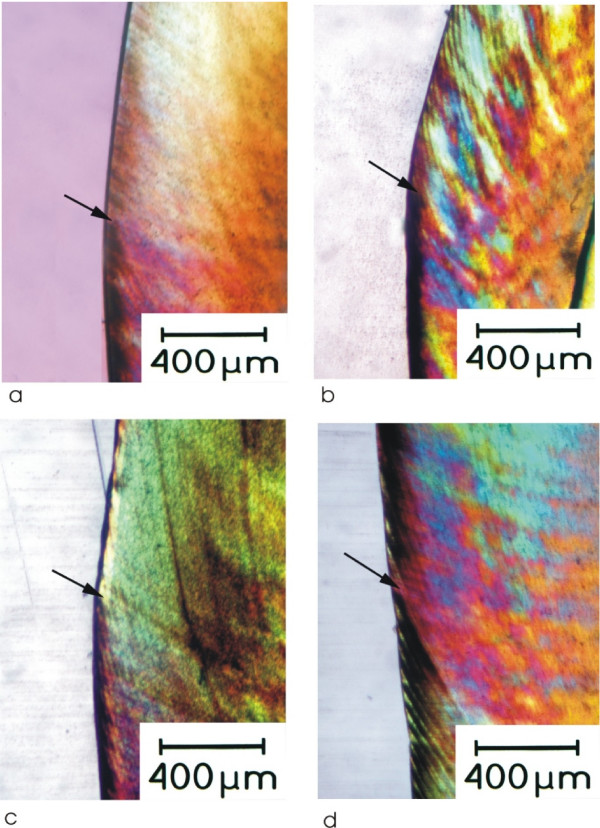
Polarization light micrographs of experimental lesions incubated with different toothpaste slurries. The arrow marks the border between the cervical control window and the occlusal experimental window. a) = incubation with Elmex Anticaries. The occlusal window shows single porosities in the body of the lesion; b) = incubation with Elmex Sensitive. The occlusal window shows an inhomogehous body of the lesion; c) = incubation with Colgate GRF. The occlusal window shows a homogeneous body of the lesion; d) = incubation with Blend-a-med complete. No differences are seen between the occlusal and cervical window.

Morphological analysis of the sections with PLM in the control windows showed interrupted bands or inhomogeneous lesions whereas in the experimental windows of the teeth treated with Elmex Anticaries, Elmex Sensitive and Colgate GRF the lesions were absent or expressed as single porosities (Table 2). Only in the teeth treated with Blend-a-med Complete two lesions were found expressed as interrupted bands and inhomogeneous with no signs of remineralization (Fig. 1). The difference between the control window and the experimental window was significant with a p value of 0.008 in the Elmex Anticaries, Elmex Sensitive and Colgate GRF samples but not in the Blend-a-med complete samples (p = 0.56).

**Table 2:** Number of lesion categories and morphological code numbers in the different groups.

Group/code	Morphology of the body of the lesion									
	
	Control window					Experimental window				
	
	1	2	3	4	5	1	2	3	4	5
Placebo toothpaste			1	4				1	4	
Remineralization solution			1	3	1			1	3	1
Elmex Caries Protection			2	2	1	1	4			
Elmex Sensitive			2	2	1	2	3			
Blend-a-Med Complete			1	4		2	1	1	1	
Colgate GRF			3	2		2	3			

Quantitative element analysis revealed no statistically significant differences in the content of P, C and F between any of the groups in the body of the lesion of the control and experimental windows. A statistically significant difference of the Ca content in the superficial enamel layer between the experimental window and the control window was found in the Elmex Anticaries group with a higher Ca content in the experimental window. No difference was found in the Ca/P ratio in any of the groups and windows.

Acid etching of the superficial enamel layer showed an increased fluoride content in the Elmex Anticaries, Elmex Sensitive and Blend-a-med Complete groups compared to the control areas. No difference was found between the experimental window and the control area in the Colgate GRF and remineralization group and the control group (Table 3). Acid resistance measured in terms of P solubility was highest in the Elmex Anticaries group, whereas the lowest acid resistance with no difference to the control group was found in the Colgate GRF group (Table 4).

**Table 3:** Fluoride content of the superficial enamel layer after treatment with different toothpaste slurries.

Group	n	Fluoride content in mg/7.1 mm^2^	P value
Placebo tooth pastel	10	26.33 ± 5.12	Ø
Remineralization solution	9	20.81 ± 3.42	> 0.05
Elmex Anticaries	10	58.11 ± 8.23	< 0.001
Elmex Sensitive	10	107.40 ± 23.11	< 0.001
Blend-a-med Complete	10	66.82 ± 16.39	< 0.001
Colgate GRF	9	35.84 ± 7.75	> 0.05

**Table 4:** Phosphorus solubility of the superficial enamel layer after treatment with different toothpaste slurries.

Group	n	Phosphorus content in mg/l	P value
Placebo tooth paste	10	21.89 ± 1.93	Ø
Remineralization solution	9	20.27 ± 2.24	> 0.05
Elmex Anticaries	10	15.13 ± 2.55	< 0.001
Elmex Sensitive	10	16.9 ± 2.25	< 0.001
Blend-a-med Complete	10	16.18 ± 2.58	< 0.001
Colgate GRF	9	21.46 ± 3.42	>0.05

## Discussion

Demineralization of enamel leads to dissolution of hydroxyapatite and diffusion of Ca and P ions towards the enamel surface. Hypersaturation of Ca and P ions on the surface results in a re-precipitation of hydroxyapatite forming the intact superficial layer on the enamel surface. Remineralization of enamel is enhanced by the presence of fluoride ions [17,27]. The morphology of the body of the lesion and the superficial enamel layer reflects its mineralization and can be determined by its birefringence with PLM [28]. The morphological results of this study indicate that application of amine fluoride and sodium monofluorophosphate results in complete remineralization of the body of the lesion or in single porosities. Acid solubility of enamel is reduced after amine fluoride application. This leads to the conclusion, that amine fluoride not only enhances enamel remineralization but also results in a more stable, less soluble superficial enamel layer.

Bioavailability of fluoride from dentifrices and the question of systemic or topical action of fluoride has long been the subject of scientific discussion. The efficiency of ionic fluoride from toothpastes has been proved in recent in vitro studies [7,12,21,29], supported by in vivo studies [9,30]. Salivary clearance of fluoride has been discussed with controversial results. Although salivary fluoride content after tooth brushing with fluoride containing toothpastes decreases significantly after mouth rinsing with water, amine fluoride showed significantly higher salivary fluoride levels 90 min after brushing than NaF [31].

Although there are clear advantages of morphological methods they have limitations which are the variability of the lesions and the individual judgment of the investigator. However, within the limits of these methods it could be shown that all dentifrices enhanced enamel remineralization of the experimental lesions. The morphology of the lesions differed slightly between the groups. After amine fluoride application a more homogenous body of the lesion was found which may be explained by the slow release of fluoride and a more constant salivary fluoride level.

From a theoretical point of view the superficial remineralized enamel layer contains fluoroapatite because at a pH between 4.5 – 5.0 fluoroapatite is supersaturated, contributing to remineralization, whereas hydroxyapatite is undersaturated contributing to demineralization [1]. The fluoride and phosphorus measurements of this investigation show a significantly higher fluoride content and lower phosphorus solubility in the superficial layer which may be due to CaF_2 _precipitation on the enamel surface because phosphorus incorporated into CaF_2 _is less soluble [32]. The different fluoride compounds result in an increased fluoride uptake in the superficial enamel layer, but not in the former body of the lesion. The remineralization of the body of the lesion appears to be more homogeneous after application of amine fluoride and sodium monofluorophosphate. These results are confirmed by the EDX measurements demonstrating no differences in the element content to sound enamel.

## Conclusion

It can be concluded that all fluoride compounds enhanced enamel remineralization in this in vitro experiment in the following order: NaF < sodium monofluorophosphate < amine fluoride. The superficial enamel layer seems to be more stable after amine fluoride application than after sodium fluoride and sodium monofluorophosphate application.

The morphological approach clearly demonstrates the complete remineralization of the experimental caries like lesion in 7 out of 20 samples due to amine fluoride, sodium fluoride and sodium monofluorphosphate availability, and the incomplete remineralization in other 11 lesions. Only 2 lesions from the NaF group showed no changes to the demineralized control. Serial sections through all experimental and control caries like lesions supported by SEM and EDX element analysis give a comprehensive feature of the effectiveness of different fluoride availability simulating a longer tooth brushing period to prevent demineralization of subsurface lesions and contributing to remineralization of PLM detected mineral loss.

## Competing interests

This study has been supported by GABA International, Münchenstein, Switzerland.

The authors declare that they have no financial competing interests, as there have been no conditions from this support concerning the publication of the results.

## Authors' contributions

WHA was the supervisor of the project and responsible for the manuscript draft.

AD carried out the PLM investigations

SL carried out the EDX measurements

ZG carried out the slurry experiments and F measurements

JB supported the experiments of ZG and contributed to the manuscript draft

PG contributed to the planning of the project, evaluation of the results and writing of the manuscript

## Pre-publication history

The pre-publication history for this paper can be accessed here:


